# Pretreatment organ function in patients with advanced head and neck cancer: clinical outcome measures and patients' views

**DOI:** 10.1186/1472-6815-9-10

**Published:** 2009-11-15

**Authors:** Lisette van der Molen, Maya A van Rossum, Annemieke H Ackerstaff, Ludi E Smeele, Coen RN Rasch, Frans JM Hilgers

**Affiliations:** 1 Department of Head and Neck Oncology & Surgery, The Netherlands Cancer Institute, Amsterdam, The Netherlands; 2 Department of Ear, Nose, Throat, Leiden University Medical Centre, Leiden, The Netherlands; 3 Department of Radiation Oncology, The Netherlands Cancer Institute, Amsterdam, The Netherlands; 4 Institute of Phonetic Sciences, University of Amsterdam, Amsterdam, The Netherlands; 5 Academic Medical Centre/University of Amsterdam, Amsterdam, The Netherlands

## Abstract

**Background:**

Aim of this study is to thoroughly assess pretreatment organ function in advanced head and neck cancer through various clinical outcome measures and patients' views.

**Methods:**

A comprehensive, multidimensional assessment was used, that included quality of life, swallowing, mouth opening, and weight changes. Fifty-five patients with stage III-IV disease were entered in this study prior to organ preserving (chemoradiation) treatment.

**Results:**

All patients showed pretreatment abnormalities or problems, identified by one or more of the outcome measures. Most frequent problems concerned swallowing, pain, and weight loss. Interestingly, clinical outcome measures and patients' perception did no always concur. E.g. videofluoroscopy identified aspiration and laryngeal penetration in 18% of the patients, whereas only 7 patients (13%) perceived this as problematic; only 2 out of 7 patients with objective trismus actually perceived trismus.

**Conclusion:**

The assessment identified several problems already pre-treatment, in this patient population. A thorough assessment of both clinical measures and patients' views appears to be necessary to gain insight in all (perceived) pre-existing functional and quality of life problems.

## Background

In recent years chemoradiation (CRT) has become an indispensable treatment modality for advanced head and neck cancer, improving local control and overall survival in all sites, except in the (stage IV) laryngeal cancers [1-3]. Unfortunately, CRT can have a detrimental effect on organ function, and on patients' quality of life [4]. But even before the onset of treatment patients may present with pain, impaired swallowing, aspiration, dietary restrictions and even with tube dependency, as well as trismus and loss of body weight, because the tumor may disrupt the normal anatomy and thus interfere with normal function [5-11]. Many studies have indeed investigated typical problems associated with head and neck cancer [12-16]. However, the majority of earlier research has focused on posttreatment dysfunction [4]. Moreover, the studies investigating problems associated with head and neck cancer tend to focus on only a limited set of functional aspects.

A systematic search of the literature, covering the period between January 1997 to August 2007 [4], shows that two assessment tools are commonly used in the literature. Quality of life (QOL) questionnaires are frequently used to evaluate patients' perceived quality of life -where other functional outcomes such as pain and nutrition are generally part of the QOL questionnaire [4] -, or Videofluoroscopic Modified Barium Swallow (VMBS) examinations are used to assess swallowing function [4]. VMBS examinations only assess the structures and dynamics of the swallowing process, and do not assess the influence of the swallowing problems on the patients' overall quality of life (personal perception of well-being). In the literature only a few studies combine VMBS examinations with QOL questionnaires [6-8,11]. These studies also show some limitations; i.e. the VMBS was only performed after CRT and based on patient or clinician appreciated swallowing difficulties beyond that expected after treatment [7]; the authors did not use a QOL questionnaire to analyze the patients' perceived problems, but only one single question [8] or a 7-point scale [11]; or the authors did not correlate the VMBS examinations to the QOL outcomes [6].

As mentioned above, the problems associated with head and neck cancer may involve many different functional aspects, most of which deteriorate even further through CRT. It is therefore imperative that a comprehensive multidimensional assessment is applied to identify existing problems before onset of treatment and monitor these problems during and post-treatment. This not only provides important baseline measurements to evaluate the effects of e.g. preventive and/or therapeutic rehabilitation programs, but also allows thorough analysis and comparison of the subjective patient-perceived and objective clinician-measured treatment outcomes.

The aim of this study is to evaluate the pretreatment organ function in advanced head and neck cancer patients through patients' view and clinical measures. The correlations between, and the importance of the different subjective patient-perceived as well as objective clinician-measured aspects are reported in this study.

## Methods

During the accrual period (September 2006-April 2008) of this study 72 patients were treated with CRT for advanced head and neck cancer. Seventeen patients could not be included, because of patient refusal (N = 4), follow-up known in advance to be abroad (N = 2), administrative miss (N = 1), cognitive problems (N = 6), or physical problems (N = 4, i.e. Bechterew's disease, tetraplegia, jaw problems), leaving 55 patients (76%) for inclusion in the study. All 55 patients with a known primary tumor (advanced stage III and IV squamous cell carcinoma) of the oral cavity, oropharynx, hypopharynx, larynx and nasopharynx, participated in this study. All patients were eligible for treatment with chemoradiation with curative intent and were referred to the Netherlands Cancer Institute for their primary treatment. Data were collected as baseline measurements for a Randomised Clinical Trial (RCT) on "Prevention of trismus, swallowing and speech problems in patients treated with chemoradiation for advanced head and neck cancer". The medical ethical review board of the Institute approved the study protocol, and written informed consent was obtained from all patients before they entered the study. The study group consisted of 44 males and 11 females, with a mean age of 58 years (range 32-79 years). Patients' characteristics, including tumor sites and stages, are shown in Table 1. Staging was according to the International Union against Cancer (UICC), 5^th ^edition, 2005. The majority of the patients (N = 38; 69%) had stage IV disease; 17 patients (31%) had stage III.

**Table 1:** Patient characteristics (N = 55)

Characteristic	Finding	(%)
Age in years		
Mean	58	
Range	32-79	

Sex		
Male	44	(80)
Female	11	(20)

T category		
T1	8	(15)
T2	15	(27)
T3	21	(38)
T4	11	(20)

N category		
N0	6	(11)
N1	15	(27)
N2	28	(51)
N3	6	(11)

Stage		
III	17	(31)
IV	38	(69)

Tumor sites		
**Oral cavity**	**5**	**(9)**
Floor of mouth	2	(4)
Tongue	3	(5)
**Oropharynx**	**24**	**(44)**
Retromolar trigone	1	(2)
Base of tongue	10	(18)
Tonsil	7	(13)
Soft palate	2	(4)
Pharynx posterior wall	3	(5)
Valleculae	1	(2)
**Laryngo/hypopharynx**	**19**	(**35)**
Piriform sinus	17	(31)
Hypopharynx posterior wall	1	(2)
Supraglottic larynx	1	(2)
**Nasopharynx**	**7**	**(13)**

### Assessment aspects

The outcomes that were assessed concerned quality of life, as well as functional aspects such as nutrition, pain, swallowing, mouth opening, and weight changes. The average time to complete the total comprehensive multidimensional assessment was 90 minutes.

#### Quality of life, nutrition, pain

Quality of life was assessed by a Dutch study specific questionnaire, which includes detailed and symptom-specific questions relevant for this specific cancer group. Furthermore, earlier studies in our institute using this study specific questionnaire showed its validity [17-19] and did not reveal any differences when compared to validated standardized questionnaires such as (QLQ-C30 and QLQ-H&N35). The advantage of the present questionnaire is that more specific function-related questions could be included. This is an important consideration, because truly function-specific questionnaires such as the MDADI and the Swalqol were not yet available in Dutch at the start of this research project [20,21]. The questionnaire was completed by the patients themselves in the presence and often with assistance of the first author (LM). Nutrition was evaluated by 1 question of the study specific questionnaire (see Additional file 1: b4) and using the Functional Oral Intake Scale (FOIS). The FOIS is a validated and reliable tool, that consists of a 7-point ordinal scale, ranging from 1 (nothing by mouth), to 7 (total oral diet with no restrictions) [22]. Additionally, pain (in the head and neck region) was assessed using the commonly used, reliable visual analogue scale (VAS) of 100 mm, where pain between 0-4 mm represents no pain, pain between 5 mm and 44 mm mild pain, pain between 45-74 mm moderate pain, and severe pain was scored when a pain score between 75 to 100 mm was given [23].

#### Swallowing

Chewing, swallowing function, swallowing frequency, and the use of drinks during the meal to ease food down (all patients' perception) were assessed using 9 study specific structured questions (see Additional file 1: questions b7-b15).

The clinician-measured swallowing function was evaluated through videofluoroscopy (VFS) using the KAY swallowing workstation (Kay Elemetrics/Pentax, Lincoln Park, NY, USA). Videofluoroscopy was preferred to, for instance, FEES [24], because it allows examination of movement patterns of the bolus and of particular structures in slow motion and frame by frame. VFS studies provide information on bolus transit times, motility problems, and amount, and, most important, etiology of aspiration [25]. All patients were asked to swallow different consistencies of varying amounts twice (1 and 5 cc thin liquid; 3 cc paste; as well as solid (Omnipaque coated cake) pretreatment (comparable to protocols used by others [8,25]). The ability of the oral cavity and pharynx to move food efficiently and safely into the esophagus was assessed using the Penetration and Aspiration Scale (PAS) and an overall 'presence of residue' score. The PAS is a tool with an acceptable reliability, and consists of an 8-point scale, ranging from 1 (material does not enter the airway), to 8 (material enters the airway, passes below the vocal folds, and no effort is made to eject) that describes the depth of aspiration of the swallowed bolus into the airway [26]. For all consistencies, the first swallow was used for analysis, both for the PAS and the presence of residue. The presence of residue was scored as 'no residue', 'residue above the valleculae' (included 1. the lateral sulcus or floor of the mouth and/or 2. valleculae (minimal residue is judged as normal [5,25]); 'residue below the valleculae' (included 3. posterior wall of the pharynx and/or 4. pyriform sinuses) and 'residue above and below the valleculae'. Each videofluoroscopic study was reviewed in real-time, slow motion, and frame-by-frame. All swallow studies were scored by the first author (LM), and several days later, twenty percent of the videos were scored again to determine intraobserver reliability of PAS and overall presence of residue, which were .93 and .93, respectively. Twenty percent of the tapes were also scored by another experienced speech language pathologist to determine inter-observer reliability of PAS and overall presence of residue, which were .98 and .74, respectively.

#### Mouth opening

The patients' perceived (subjective) mouth opening was assessed by the specific question: "How do you experience your mouth opening?" and by two questions on whether there are problems caused by a possible limited mouth opening while eating and speaking (see Additional file 1: questions b3, b5, b6).

The Maximal Interincisor Opening (MIO) of the mouth was measured by the clinician using the TheraBite range-of-motion scale (Atos Medical, Hörby, Sweden). Since the chemoradiation could cause pain in the mouth, xerostomia, and/or edema, which could prevent patients from wearing dentures, mouth opening was measured without dentures (total or partial, depending on the patient). Dijkstra et al. (2006) did not find a clear cut-off point for the subgroups dentate, partially dentate and edentulous, but a mouth opening of 35 mm or less was regarded as the cut-off point for trismus of the total group [27]. Therefore, in this study a cut-off point of 35 mm or less was taken as the threshold for the total group.

#### Weight changes

Weight loss and weight gain was measured at the start of treatment and followed-up. As a prognostic factor, the average weight of the last six months pretreatment indicated by the patients themselves was compared to the measured weight at the start of chemoradiation treatment [10]. Also the clinical prognostic factor Body Mass Index (BMI) was analyzed, because a recent study by McRackan et al (2008) suggests that chemoradiation patients with increased Body Mass Index (BMI; > 25 kg/m^2^) have improved swallowing outcomes, longer time to disease recurrence, and improved survival when compared to similar patients with lower BMI.

### Statistical Analysis

All statistical analyses were performed in SPSS version 15 (SPSS, Inc, Chicago, Illinois). Descriptive statistics were used to characterize the sample. Categorical variables were compared using the χ^2 ^test. Pearson's correlation coefficient was used to investigate relationships among the different aspects of assessment. The cut-off for a meaningful level of correlation was taken as 0.3. Trial specific items of the questionnaire were combined into a more limited set of multiple-item Likert's scales. The reliability of the scales was assessed with Cronbach's alpha. For all analyses, a P < .05 was considered statistically significant. Inter -and intra observer reliability was calculated using Cohen's kappa.

## Results

### Quality of life questionnaire, nutrition, pain

The study specific questionnaire provided insight into pretreatment organ function, as perceived by the patients. Good to acceptable reliability coefficients (Cronbach's alpha) were found (Table 2), indicating that a good to reasonable internal consistency of the set of items in the different subscales was achieved.

**Table 2:** Reliability coefficient (Cronbach's alpha) of the Likert scales measuring several quality of life issues (see Additional file 1 for the corresponding questions)

		No. of items	Corresponding questions	Alpha
Functional	Swallowing	6	b. 8,9,10,11,12,13	0.763
Psychosocial	Social contacts	3	c. 1,2,3	0.636

The overall rating for the social contacts (inviting/visiting/phoning family/friends) was good for the majority of patients. Forty four patients (80%) regularly invited family or friends to their home and only 11 (20%) did so just once per month or not at all. The number of patients that had a good contact with others and did not feel restricted in their social contacts was 53 (96%) and 41 (75%), respectively.

Smell and taste was scored 'poor' to 'moderate' in 9 (16%) and 13 (24%) of the patients, respectively. The correlation between smell and taste was moderate (R = .566; P < .01), i.e. patients, who reported a disturbance of taste, also experienced a significantly poorer sense of smell.

Analyzing nutrition, 44 patients (80%) scored the maximum value 7 (N = 42; oral diet with no restrictions) or value 6 (N = 2; oral diet with multiple consistencies without special preparation, but with specific food limitations) on the Functional Oral Intake Scale (FOIS). Three patients (5%) were tube dependent at the beginning of the treatment. These 3 patients mentioned that they combined tube-feeding with some attempts or with consistent oral intake of food or liquid (scale value 2). The remaining 8 patients had an oral diet with restrictions (scale values 4 and 5).

With regard to pain as measured by VAS there were 24 patients (44%) who reported no pain (0-4 mm) at the start of the treatment. Twenty three patients (42%) reported pain less than 44 mm (mild pain). Seven patients (13%) reported pain between 45 mm and 74 mm (moderate pain) and one patient experienced severe pain (2%). Most patients related the pain to the tumor. Patients with stage III tumors reported more pain than the patients with stage IV and patients with a tumor in the oropharynx reported more pain than the other groups, but these differences were statistically not significant.

### Swallowing

Six of the 55 patients (11%) complained about mastication difficulties. As shown in Table 3, the most frequent complaints related to the swallowing function concerned difficulties with the oral phase of solid food (n = 5), and the pharyngeal phase of solid food (n = 10). The reliability of a summary scale of the 6 swallowing function items, measured by Cronbach's alpha, was good (0.763). Furthermore, 23 patients (42%) reported that they had to swallow more than twice to ingest various food consistencies and 27 patients (49%) had to use water to swallow the food.

**Table 3:** Rating of swallowing (dys)function, in terms of oral and pharyngeal transport, according to the study specific questionnaire (Additional file 1: question b8-b13)

N = 55	Not at all	A little	Quite a bit	Very much
Oral transport				
Solid food	39	11	4	1
Soft (pureed) food	50	4	1	-
Liquids	53	2	-	-
Swallowing				
Solid food	27	18	6	4
Soft (pureed) food	46	8	1	-
Liquids	48	6	1	-

The videofluoroscopic studies identified seven patients (13%) with laryngeal penetration and three (5%) with overt aspiration before treatment. For the consistency of 1 cc thin liquid approximately one-third of the patients (38%) showed more than normal residue above the valleculae. Presence of residue after 5 cc thin liquids appeared most frequently (86%) above and below the valleculae. For the paste consistency, twenty three patients (42%) showed more than normal residue above the valleculae, and twenty three patients (42%) showed residue above and below the valleculae after swallowing Omnipaque coated cake. Overall, only 1 patient (2%) did not show more than normal residue on any of the consistencies or amounts. This was one of the seven patients with a nasopharyngeal tumor. No significant correlations between the Penetration and Aspiration Scale (PAS) or presence of residue and tumor location or tumor stage were found.

### Mouth opening

Of the total group, 34 patients (62%) were dentate, 8 patients (15%) were partially dentate, and 13 patients (24%) were edentulous. Patients' perceived trismus, assessed by 1 question (Additional file 1: question b3) was reported to be a problem by 7 patients (13% of the total group). This perceived presence of trismus was highly correlated with the other 2 questions concerning the relation between limited mouth opening and difficulties while eating and speaking (R = .684; P < .01) (Additional file 1: questions b5, b6)

Clinician measured mean Maximal Interincisior Opening (MIO) of the dentate patients was 45 mm (range 26-66), for the partially dentate patients it was 50 mm (range 36-65) and for the edentulous patients it was 60 (range 48 to 69). The mean MIO of the total group was 50 mm (range 26-69). Women had a mean MIO of 45 mm (range 26-67) and men of 51 mm (30-69). These differences were statistically not significant. Trismus as measured by the clinician was established in 5 patients (9%) of the total group (two women and three men). The smallest mean MIO was measured in the group of patients with an oropharyngeal carcinoma, but there was no significant correlation with tumor site. Also, no significant correlation existed between MIO and tumor stage or dentition.

### Weight changes

The average body weight 6 months prior to treatment, as indicated by the patients, was 78.4 kg (range 50-116 kg). The mean weight measured at the start of the chemoradiation treatment was 77.0 kg (range 50.0-108.0 kg), which indicates an average weight loss of 1.3%. However, two categories of patients can be distinguished in this respect, i.e. patients with or without weight loss. In the first category, there were twenty six patients (47%), who suffered from a weight loss of 7% (mean 5.5 kg, range 0.3-19.4 kg), whereas in the second category there were two patients (4%), who had no weight change, and 27 patients (49%), who gained weight (2.84%, mean 2.2 kg, range 0.2-9.5 kg) in the preceding 6 months. These two groups did not show significant differences with respect to sex, tumor location or tumor stage.

The average BMI at the start of treatment was 25.0, with 29 patients (53%) having a BMI of 25 or less. No significant difference was found with respect to sex. A significant, but weak correlation was found between tumor location and BMI (R = .394; P < .01), i.e. a BMI of 25 kg/m^2 ^or lower occurred significantly more often in patients with a tumor in the oral cavity or oropharynx, while a BMI higher than 25 occurred significantly more often in patients with a tumor in the hypopharynx/larynx or nasopharynx. No other statistically significant differences were found.

### Relationship and relevance of the different assessment tools

**Figure 1:**
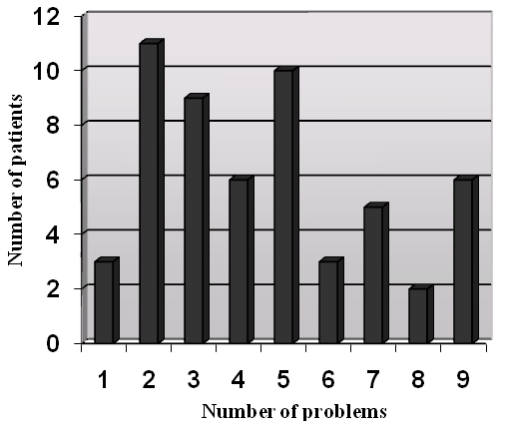
**Summary of frequencies of problems detected by the multidimensional assessment from Table 4**.

A summary of the results, given separately for each assessment aspect is presented in Table 4. A mark (X) indicates that an abnormality or problem was found (either by the clinician or perceived by the patient). All patients had at least 1 abnormality or problem, and 41 patients (75%) had 3 or more abnormalities or problems (see Figure 1). The presence of residue, perceived pain and perceived swallowing dysfunction were the most common problems.

**Table 4:** Overview of all multidimensional assessment categories per patient; a mark (X) indicates that an abnormality or problem was perceived or measured in that specific category for that patient.

	PATIENTS' PERCIEVED MEASUREMENTS						'OBJECTIVE' CLINICIAN MEASUREMENTS				
	**Qualityof life questionnaire**				**Intake**	**Pain**	**Videofluoroscopy**		**Mouth opening**	**Weight**	

**Pt.n**	**Taste/smell**	**Swallowing**	**Trismus**	**Intake**	**FOIS**		**PAS**	**Residue**	**Trismus**	**Weight loss**	**BMI < 25**

1		X		X	X	X		X			
2	X	X				X	X	X		X	X
3		X				X	X	X		X	
4	X	X	X			X		X	X	X	X
5		X		X	X			X			
6								X			X
7	X							X			
8		X		X	X	X	X	X	X	X	X
9		X		X	X	X		X		X	X
10								X			
11	X	X		X	X	X		X		X	X
12	X					X	X	X			X
13		X						X			
14		X				X		X	X		
15		X				X		X			X
16						X		X		X	
17								X			X
18						X		X			
19	X	X		X	X			X			X
20	X	X						X			
21								X			
22	X	X	X				X	X		X	X
23		X				X		X		X	
24								X		X	
25								X			
26								X			X
27		X	X	X	X	X		X			X
28		X		X	X		X	X			X
29	X	X		X	X	X	X	X		X	X
30		X	X								
31		X				X		X			
32	X	X		X	X			X		X	X
33	X							X			X
34		X				X		X			
35								X			X
36	X	X				X		X			X
37		X				X		X		X	X
38								X			
39	X	X	X			X		X		X	
40							X	X		X	
41		X				X		X		X	
42		X				X		X		X	X
43	X	X		X	X			X		X	X
44		X				X		X			
45								X	X	X	
46	X					X		X		X	X
47			X			X		X		X	X
48	X							X			
49		X				X		X			
50		X	X			X	X	X	X	X	X
51							X	X			X
52	X	X						X			X
53		X				X		X		X	X
54		X				X		X		X	
55						X		X			X

Discrepancies exist when comparing the patients' perceived problems with the problems as identified by the clinician. Three of the 10 patients (30%), who penetrated or aspirated as judged on the PAS, did not report any swallowing problems. Conversely, of the 12 patients who reported perceived swallowing problems only 5 patients showed laryngeal penetration or aspiration (42%). However, the difference between the PAS and the patients' perceived swallowing problems was not significant.

Trismus measured by the clinician occurred, as already mentioned, in 5 patients and subjectively, trismus was perceived by 7 patients (13% of the total group), but in only 2 of the 7 patients a restricted mouth opening (< 35 mm) was measured by the clinician. The 3 other objective trismus patients did not report having a limited mouth opening. Thus, patients with a restricted mouth opening did not necessarily perceive a limited mouth opening, and some patients with a 'normal' mouth opening (> 35 mm), did perceive limited mouth opening. The negative correlation between the clinician measured and the patients' perceived trismus was significant but weak (R = -.359; P < .01).

A statistically significant correlation was found between the clinician-rated FOIS and the question 'diet' of the quality of life questionnaire (R = -.962; P < .01). Patients who combined tube-feeding with some attempts or with consistent oral intake of food or liquid (FOIS scale value 2) had also answered the question 'diet' with 'combination soft diet and tube feeding' (Additional file 1; question b4; value 5).

A (weak) significant correlation was further found between weight and FOIS (R = .346; P = .023): patients who were tube dependent lost more weight than patients who had no oral restrictions.

## Discussion

This study of 55 patients with advanced (stage III and IV) squamous cell carcinoma of the oral cavity, oropharynx, hypopharynx, larynx or nasopharynx, shows that in view of the many abnormalities and problems found and the lack of positive correlations between the various outcome instruments, a multidimensional assessment package is indispensable to evaluate organ function prior to treatment.

Several abnormalities or problems were found by the clinician or experienced by the patient. All patients had at least 1 problem and 41 patients (75%) had 3 or more abnormalities or problems. The most common problems perceived by the patients were swallowing and pain. Almost two-third of the patients experienced slight swallowing problems, and 20% had to modify their diet because of these problems. These findings are in accordance with the literature. Many studies reported pretreatment swallowing restrictions assessed by a (quality of life) questionnaire [7,8,11,28]. It is generally accepted that dietary modification should be the initial approach for patients with swallowing problems [29]. Pain is very rarely explicitly reported pretreatment and comparable data are lacking in the literature [4]. This is unfortunate, because scoring perceived pain (for which a visual analogue scale is an effective and easy method) is an important parameter in the clinical follow-up of any cancer treatment [30].

The most common problems found by the clinician were the presence of residue visualized during videofluoroscopy, and weight loss. Presence of residue appeared in almost all patients for almost all consistencies. These findings are comparable with a study by Pauloski et al. [5]. These authors also found greater amounts of oral and pharyngeal residue before treatment in head and neck cancer patients compared with normal control subjects. Although the inter-observer reliability of residue was relatively low (0.74), and its clinical relevance is unclear, this aspect of the videofluoroscopy assessment is still relevant as baseline information with which posttreatment results should be compared. Nevertheless, the subjective nature of this scale must be kept in mind. Weight at the onset of therapy, and especially weight loss in the preceding 6 months and during treatment, are strong predictors for overall and disease specific survival in head and neck cancer patients [31]. In the present study, a weight loss of 7% was calculated in half of the patients, whereas the other half showed no weight change or even gained weight. These figures have to be interpreted with caution since they are partly based on the patient's memory. Furthermore, patients were instructed to increase their oral intake directly after the first consultation, which means that they might have gained some weight by the time the treatment started. Only a few authors have reported on pretreatment body weight and even fewer on pretreatment body weight changes, despite the fact that these changes have prognostic relevance [31].

Body Mass Index (BMI) seems to be an important consideration when predicting the likely outcomes of concurrent chemotherapy for head and neck cancer patients, as recently reported by McRackan et al. [32]. These authors found an average BMI of 24.3, and a better treatment outcome for the patients with a BMI > 25. The average BMI of 25.0 found in the present study is comparable to the one reported by McRacken. However, it remains to be seen whether BMI in the present study population is a strong predictor of treatment outcome as well, and whether an actual overweight improves the therapy outcome. For the moment it does seem worthwhile to include BMI as part of a thorough assessment.

The multidimensional assessment in this study identified several functional problems pretreatment. Furthermore, several problems objectified by the clinician were not perceived as such by patients. For instance, 3 of the 10 patients (30%) who showed laryngeal penetration or aspirated did not report any swallowing problems, which is alarming. These patients have an increased risk of aspiration pneumonia and should be monitored very closely during treatment as well as start with intensive rehabilitation before treatment. If only a questionnaire had been used, this 'risk' would not have been found and possible negative consequences could not have been prevented. Studies that do not combine VMBS examinations with QOL questionnaires may not identify all relevant functional problems, and therefore, the care these patients need may be lacking. Another important reason to start intensive rehabilitation before treatment is the significant evidence that pretreatment swallowing exercises do improve post-treatment swallowing function in head and neck cancer [33].

## Limitations of the study

The multidimensional assessment used in this study is not standard. As far as we know, there is no standard assessment battery that includes all the different aspects that could be affected in this population. We were therefore forced to compile an evaluation package, which is described in this study. There are other assessment tools available, however, one has to choose as: it is obviously not realistic to include every available assessment tool. For our research project we endeavored to find a balance between thorough, comprehensive evaluation and burden to the patient.

## Conclusion

Considerable functional problems and abnormalities exist pretreatment in patients with advanced head and neck cancer. The comprehensive multidimensional assessment used in this study provides good baseline information for later evaluation of functional outcomes of the oncologic treatment and/or rehabilitation. Additionally, this study clearly shows that not only clinical outcomes measures should be included, but that patients' views should also be collected in order to gain good insight in all pre-existing functional and quality of life problems in these patients.

## Competing interests

Part of the study is supported by an unrestricted research grant from Atos Medical, Hörby, Sweden.

## Authors' contributions

LM coordinated the study, collected the data, performed the statistical analyses and drafted the manuscript. MR and FH designed the study, analyzed and interpreted the data, and revised the manuscript critically. AA participated in the design of the study, especially with respect to the study specific questionnaires, and revised the manuscript critically. LS and CR participated in the design of the study, and revised the manuscript critically. All authors read and approved the final manuscript.

## Pre-publication history

The pre-publication history for this paper can be accessed here:

http://www.biomedcentral.com/1472-6815/9/10/prepub

## Supplementary Material

Additional file 1 **Selection of the translated Dutch study specific questionnaire**. This file represents a selection of the translated Dutch study specific questionnaire, covering specific functions. Click here for file
