# Supplementary material for: Pretreatment organ function in patients with advanced head and neck cancer: clinical outcome measures and patients' views
Source: BMC Ear Nose Throat Disord. 2009 Nov 15;9:10. doi: 10.1186/1472-6815-9-10 (PMC2779790; doi:10.1186/1472-6815-9-10)
Supplement: Additional file 1 — Selection of the translated Dutch study specific questionnaire. This file represents a selection of the translated Dutch study specific questionnaire, covering specific functions. [file 1472-6815-9-10-S1.DOC]

**Additional file 1:** Selection of the translated Dutch study specific questionnaire.

**Study specific questionnaire**

1. Socio-demographic data (12 questions)
2. Complaints over the last week
   1. Sense of smell (4 questions)
      - 1. What is your smell like?

1 = bad 2 = fair

3 = good 4 = excellent

- - - 1. Has your sense of smell changed after treatment?

1 = much worse 2 = slightly worse

3 = the same 4 = a bit better

5 = much better 6 = not applicable

- - - 1. What is your taste like?

1 = bad 2 = fair

3 = good 4 = excellent

- - - 1. Has your taste changed after treatment?

1 = much worse 2 = slightly worse

3 = the same 4 = a bit better

5 = much better 6 = not applicable

- 1. Diet, swallowing and chewing (17 questions)
     - 1. Do you still have your own teeth?

1 = yes 2 = yes, partially

3 = no, I have a prosthesis 4 = no, and I don’t wear a prosthesis

- - - 1. How often do you clean your teeth?

1 = a couple of times a day 2 = once a day

3 = less than once a day 4 = not at all

- - - 1. How do you experience your mouth opening?

1 = normal 2 = a little bit limited

3 = very limited 4 = I cannot open my mouth

- - - 1. What is your diet like?

1 = I eat solid food 2 = I only eat soft (minced) food

3 = I only eat liquid food 4 = I only have tube feeding

5 = combination soft diet and tube feeding

- - - 1. Do you experience problems with eating, because of a limited mouth opening?

1 = not at all 2 = a little

3 = rather 4 = quite a lot

- - - 1. Do you experience problems with speech, because of a limited mouth opening?

1 = not at all 2 = a little

3 = rather 4 = quite a lot

- - - 1. Do you have problems with chewing your food?

1 = not at all 2 = a little

3 = rather 4 = quite a lot

- - - 1. Do you have problems with moving solid food around in your mouth?

1 = not at all 2 = a little

3 = rather 4 = quite bad

- - - 1. Do you have problems with moving soft/minced food around in your mouth?

1 = not at all 2 = a little

3 = rather 4 = quite a lot

- - - 1. Do you have problems with moving liquid food around in your mouth?

1 = not at all 2 = a little

3 = rather 4 = quite a lot

- - - 1. Do you have problems with swallowing solid food?

1 = not at all 2 = a little

3 = rather 4 = quite a lot

- - - 1. Do you have problems with swallowing soft/minced food?

1 = not at all 2 = a little

3 = rather 4 = quite a lot

- - - 1. Do you have problems with swallowing liquid food?

1 = not at all 2 = a little

3 = rather 4 = quite a lot

- - - 1. Do you have to swallow repeatedly to get rid of food?

1 = yes 2 = no

3 = sometimes

- - - 1. Do you have to drink during a meal to ease food down?

1 = yes 2 = no

3 = sometimes

- - - 1. Do you have a normal amount of saliva (spit)?

1 = much less 2 = a bit less

3 = the same 4 = a bit more

5 = much more

- - - 1. Can you keep your saliva in the mouth without leakage?

1 = not at all 2 = a bit

3 = fairly well 4 = quite easily

- 1. Social contacts (6 questions)
     - 1. How frequently did you visit family or friends over the last month?

1 = every day 2 = a few times a week

3 = once a week 4 = 2-3 times a week

5 = once this month 6 = not at all

- - - 1. How frequently did family or friends visit you?

1 = every day 2 = a few times a week

3 = once a week 4 = 2-3 times a week

5 = once this month 6 = not at all

- - - 1. How frequently did you phone family or friends over the last month?

1 = every day 2 = a few times a week

3 = once a week 4 = 2-3 times a week

5 = once this month 6 = not at all

- - - 1. How has your contact been with others, recently?

1 = bad 2 = fair

3 = reasonable 4 = good

- - - 1. Do you feel compromised in your contact with others?

1 = not at all 2 = a little

3 = rather 4 = severely

- - - 1. Do you avoid strangers?

1 = never 2 = sometimes

3 = frequently 4 = always
